# To Establish an Early Prediction Model for Acute Respiratory Distress Syndrome in Severe Acute Pancreatitis Using Machine Learning Algorithm

**DOI:** 10.3390/jcm12051718

**Published:** 2023-02-21

**Authors:** Wanyue Zhang, Yongjian Chang, Yuan Ding, Yinnan Zhu, Yawen Zhao, Ruihua Shi

**Affiliations:** 1 Department of Medical School, Southeast University, Nanjing 210009, China; 2 Department of Gastroenterology, Southeast University Affiliated Zhongda Hospital, No. 87 Dingjiaqiao, Nanjing 210009, China; 3 Department of Cyberspace Security, Institute of Southeast University, Nanjing 210009, China

**Keywords:** acute respiratory distress syndrome, acute severe pancreatitis, machine learning, artificial neural network

## Abstract

Objective: To develop binary and quaternary classification prediction models in patients with severe acute pancreatitis (SAP) using machine learning methods, so that doctors can evaluate the risk of patients with acute respiratory distress syndrome (ARDS) and severe ARDS at an early stage. Methods: A retrospective study was conducted on SAP patients hospitalized in our hospital from August 2017 to August 2022. Logical Regression (LR), Random Forest (RF), Support Vector Machine (SVM), Decision Tree (DT), and eXtreme Gradient Boosting (XGB) were used to build the binary classification prediction model of ARDS. Shapley Additive explanations (SHAP) values were used to interpret the machine learning model, and the model was optimized according to the interpretability results of SHAP values. Combined with the optimized characteristic variables, four-class classification models, including RF, SVM, DT, XGB, and Artificial Neural Network (ANN), were constructed to predict mild, moderate, and severe ARDS, and the prediction effects of each model were compared. Results: The XGB model showed the best effect (AUC = 0.84) in the prediction of binary classification (ARDS or non-ARDS). According to SHAP values, the prediction model of ARDS severity was constructed with four characteristic variables (PaO_2_/FiO_2_, APACHE II, SOFA, AMY). Among them, the overall prediction accuracy of ANN is 86%, which is the best. Conclusions: Machine learning has a good effect in predicting the occurrence and severity of ARDS in SAP patients. It can also provide a valuable tool for doctors to make clinical decisions.

## 1. Introduction

Acute pancreatitis (AP) is a common disease in gastroenterology. It is estimated that about 20% of AP patients can develop severe acute pancreatitis (SAP) with persistent organ failure and become critically ill [1,2]. Among them, acute respiratory distress syndrome (ARDS) is the leading cause of lung failure in SAP patients, and the incidence can be as high as 1/3. Respiratory death caused by ARDS accounted for 60% of all causes of death in SAP patients within one week of onset [3,4]. However, so far, the treatment options for ARDS are still limited, and the improvement of prognosis mainly depends on early identification and the use of mechanical ventilation [5]. Currently, the clinical diagnosis and severity evaluation of ARDS still rely on identifying symptoms such as dyspnea and monitoring blood gas analysis by doctors. However, the accuracy of the judgment is not satisfactory due to the intense subjectivity. To solve this problem, some scholars are trying to find new biomarkers that can predict ARDS [6], and some scholars have developed predictive scoring systems to predict the occurrence of ARDS [7]. However, these prediction models are established mainly based on traditional statistical methods. Due to the inability to consider multicollinearity between variables, the prediction ability is often uneven and cannot meet clinical needs.

Artificial intelligence machine learning technology has made significant progress in medicine in recent years. Studies have shown that machine learning has a better predictive effect than traditional statistical analysis [8], which provides an unprecedented opportunity to establish new practical ARDS prediction tools for SAP patients. 

Several previous publications have demonstrated the advantages of machine learning algorithms in ARDS prediction [9,10,11]. However, few models have used characteristic variables associated with AP, such as amylase and inflammatory markers, which may seriously affect the prediction of ARDS in AP patients. Yang et al. [12] tried to use the ANN algorithm to predict ARDS in AP patients but needed to explain the rationale behind the model, which will limit the clinical application of this model.

Therefore, this work attempted to combine machine learning methods to establish binary and quaternary classification ARDS prediction models in SAP patients and to find the model with the best prediction performance. By analyzing the clinical data of patients, this model can quickly and accurately determine the high-risk patients with ARDS and understand the severity of expiratory distress in patients, providing a valuable tool for clinical decision-making. To the best of our knowledge, this is the first validated model that applies interpretable machine learning to predict the occurrence and severity of ARDS in SAP patients.

## 2. Material and Methods

### 2.1. Data Resources and Study Cohorts

A retrospective study of patients diagnosed with SAP between August 2017 and August 2022 at Zhongda hospital was conducted using data from the electronic database. These patients were admitted within 72 h of onset, and their diagnosis of SAP met the Atlanta Classification (2012) [13] criteria: the presence of persistent single or multiple organ failure for ≥48 h. Patients with a previous history of chronic pulmonary disease; pancreatitis after ERCP, acute exacerbation of chronic pancreatitis, acute pancreatitis in pregnancy; and comorbid malignancies, autoimmune diseases, and hematological disorders were excluded.

The primary working outcome was acute respiratory distress syndrome, diagnosed and classified according to the Berlin definition [14]. There are 440 SAP patients in this research dataset, including 230 non-ARDS patients and 210 ARDS patients. The sample categories are roughly balanced. Among the 210 patients with ARDS, 40 patients with mild ARDS, 110 with moderate ARDS, and 60 with severe ARDS were included.

This study was performed in Zhongda Hospital affiliated to Southeast University. The protocol was consent to the Declaration of Helsinki and its later declarations.

### 2.2. Data Collection and Preprocessing

In contrast to existing risk-scoring models that include only a limited number of clinical features, this work considers the broadest possible range of factors based on clinically available data. Demographic data, etiology, medical history, hemodynamic parameters, laboratory index, and scoring system for disease severity were collected separately for each patient from the electronic health record. Etiologies were categorized as cholestatic, alcoholic, hyperlipidemia, and other. Medical history included some history of chronic disease and smoking and alcohol. Hemodynamic parameters, including systolic, diastolic, and mean arterial pressure, were measured on admission. Laboratory test data were taken from the patient’s first peripheral venous blood sample results within 24 h of admission. 

The most clinically accepted scoring system for the severity of disease was chosen for this work: Acute Physiology and Chronic Health Evaluation II (APACHE II) score [15] and Sequential Organ Failure Assessment (SOFA) score [16] was used to evaluate the severity of the disease. They make a quantitative evaluation of the patient’s condition through objective physiological parameters, and the final score corresponds to the severity of the disease. The worst values of physiological parameters within 24 h after admission were calculated and analyzed by APACHE II and SOFA.

The above data were processed for missing values to manage the data effectively. Outliers that do not pass the logical check are treated as missing values and handled using the lost value method. Features with missing values exceeding 10% of the sample size are discarded. Stratified means were applied to fill in missing values for features with a missing value ratio of less than 10% of the sample size. The valid data set after processing is 440 * 45. There are 440 cases in total, and each case has 43 feature variables and two predicted labels. Binary and quaternary classification labels were used to predict the occurrence and severity of ARDS, respectively.

Table 1 presents the distribution of the studied characteristics. The measurement data were expressed as median (interquartile ranges). The comparison between the two groups was performed by normal distribution and homogeneity of variance test. Independent sample t-test was used for normal distribution and homogeneity of variance, and the Wilcoxon test was used for non-normal distribution. *p* < 0.05 was considered statistically significant.

### 2.3. Model Development and Evaluation

The entire work cohort was randomly divided into a training set (70%) and a test set (30%). To prevent the problem of overfitting in the training process, this work performs model training in the form of five-fold cross-validation. A total of five accuracies were obtained, and the average value was used to obtain the model accuracy.

Machine learning predictive models and statistical analysis were constructed using the Sklearn package version 1.0.2 and Python version 3.9. This work attempts to develop predictive models with the following machine learning methods, which are currently the most advanced and powerful machine learning methods in biomedicine: Logistic Regression (LR), Random Forest (RF), Support vector Machine (SVM), Decision Tree (DT), eXtreme Gradient Boosting (XGB), and Artificial Neural Network (ANN). The LR model uses the maximum likelihood function method and gradient descent to solve the parameters to achieve the purpose of binary classification of data, which is a classical method for data classification [17]. The RF model is a decision tree based on a bagging framework, which can deal with high-dimensional feature input samples and has excellent accuracy [18]. The SVM model is a supervised learning algorithm often used to solve classification problems [19]. The DT is represented by a tree structure and can mathematically predict the best choice. The DT is often used to solve problems in biological sciences due to its ease of understanding [20]. The XGB is an optimized distributed gradient-boosting library that innovates the transformation of weak learners into strong learners, significantly increasing classification accuracy [21]. The ANN is a mathematical model that simulates neurons processing information. It has strong generalization ability and robustness and can provide a high data processing efficiency [22].

Although the LR algorithm performs well in binary classification models, it is difficult for this algorithm to competently select quaternary classification labels due to its low classification accuracy [17]. Therefore, in this work, the LR algorithm was only applied to the selection of binary classification labels, that is, to judge the risk of ARDS. Given the powerful classification function of the ANN algorithm, this work added the ANN model to quaternary classification for ARDS severity prediction.

The training set (train_x, train_y) is fed into a machine learning model, which is trained to predict the classification label based on the case feature variables. Then, the test set data test_x is input into the trained model, and the predicted label obtained by the model is compared with the actual label test_y to judge the prediction effect of the model. The Area under the Curve (AUC) was used to compare the overall discriminant ability of the models, and the optimal model was selected according to the AUC value. Combined with the evaluation indicators such as accuracy, precision, recall rate, and F1 value, the classification prediction effect of each model is analyzed and compared.

### 2.4. Model Interpretation and Optimization

In this work, the SHAPley Additive interpretation (SHAP) is used to interpret model results, a standard method for interpreting machine learning models [23]. SHAP interprets the predicted value of the model as the sum of the attributed values for each input feature, and each feature has a corresponding SHAP value that represents the contribution of the feature to predicting the risk of complications. Many of the dozens of features belong to noise, affecting the model’s classification accuracy if not filtered out [24]. Therefore, to further optimize the model, this work screens the significant features according to the SHAP value of the features based on model interpretation and uses the optimized features again for model training to improve the model’s effectiveness and reduce the influence of noise.

## 3. Result

Model Construction and Optimization

**Figure 1:**
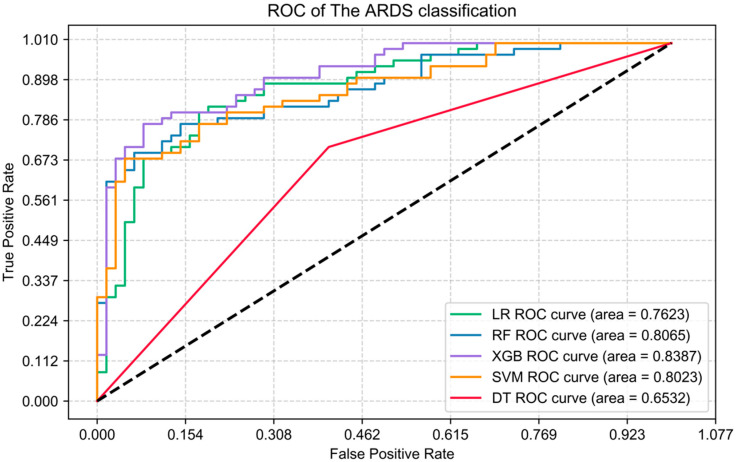
ROC curves for the five different models in the test set.

In this work, five machine learning algorithms (LR, RF, SVM, DT, XGB) were trained to predict the risk of ARDS in SAP patients (binary classification model). The training set data were randomly split into five groups, of which four groups were used for training, and the remaining one was used for testing. The other group and the remaining groups are used for validation and training. This continues until all the groups are used as the test set for testing. The average of the test results obtained each time was calculated. All of the above variables are input variables to train these five models. The receiver operating characteristic curve (ROC) showing the final prediction performance of these five models is shown in Figure 1. XGB showed the largest AUC (area = 0.839) for dichotomous variables (ARDS or non-ARDS), suggesting that it was the best predictor of ARDS. In contrast, RF showed the second highest AUC (area = 0.807) after XGB.

To further elaborate on the model performance, the evaluation metrics of accuracy, precision, recall, and F1 score (Table 2) were included in this work to compare the five models’ prediction effects. When compared, XGB showed the best predictive performance for ARDS with the highest accuracy (0.84), the highest precision (0.86), the highest recall (0.81), and the highest F1 score (0.83), while RF was the next best.

**Figure 2:**
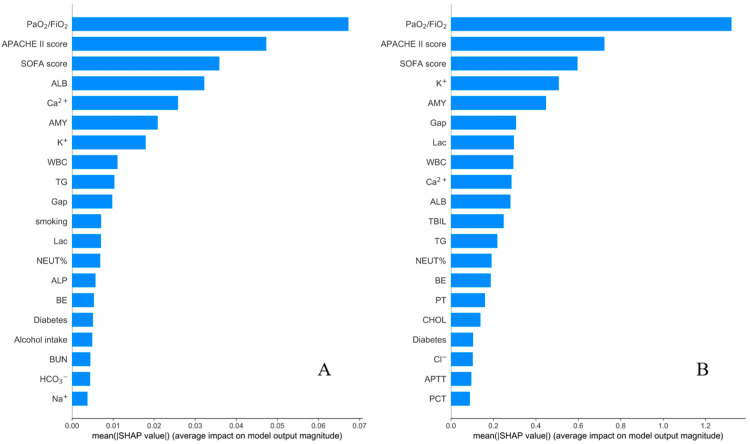
Ranking of feature importance in the Random Forest (RF) and eXtreme Gradient Boosting (XGB) models, combined with shap value. (**A**): Ranking of feature importance in the RF model; (**B**): Ranking of feature importance in the XGB model.

However, integrated learning methods such as RF and XGB, while effective, have not been able to address the issue of interpretability. On the other hand, SHAP values happen to be an effective way of interpreting the model ex-post, revealing what the model understood when it made the correct judgment. Typically, a more significant SHAP value for a feature indicates that it has a more significant impact on the model. This study, therefore, incorporates the concept of SHAP values to rank the importance of the features in the best-performing RF and XGB models to reveal the essential feature variables influencing both models (Figure 2). According to the SHAP value distribution of each feature, the crucial features that accounted for more than 80% of the importance in both models were intersected to obtain seven main features: PaO_2_/FiO_2_, APACHE II, SOFA, ALB, AMY, K^+^, and WBC, which formed the new dataset.

**Figure 3:**
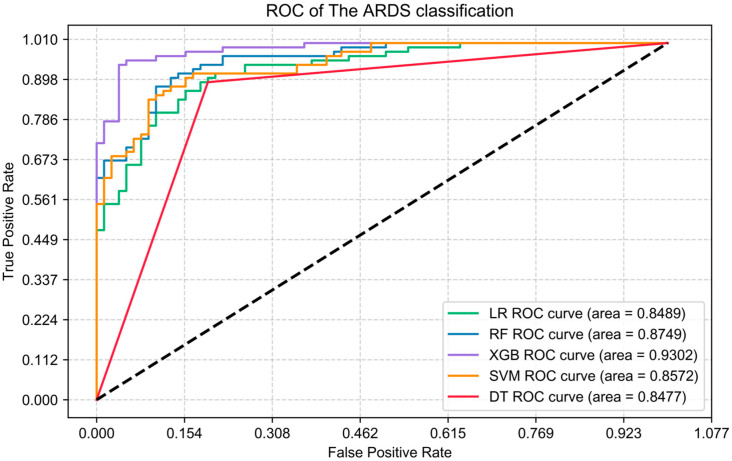
Receiver Operator Characteristic (ROC) curves of the five models after optimization.

Useless features will not help the model but will reduce the prediction effect of the model. Eliminating some irrelevant features can reduce the model’s complexity while ensuring the features’ effectiveness in improving the model’s accuracy. Therefore, this work used the data set of the seven features optimized above to train these five models again. This noise reduction process allows for more explicit sample characteristics and improved model performance. Using a number of evaluation metrics (AUC, accuracy, precision, recall, and F1 score), this work compared model performance changes in predicting ARDS before and after optimization (Figure 3). The prediction performance of all five models improved to varying degrees, with the accuracy of the LR, RF, SVM, DT, and XGB models increasing by 7%, 6%, 9%, 20%, and 9%, respectively, the AUC rising by 9%, 6%, 6%, 20%, and 9% respectively. The remaining metrics (accuracy, recall, and F1 score) also increased significantly, as detailed in Table 3. 

**Figure 4:**
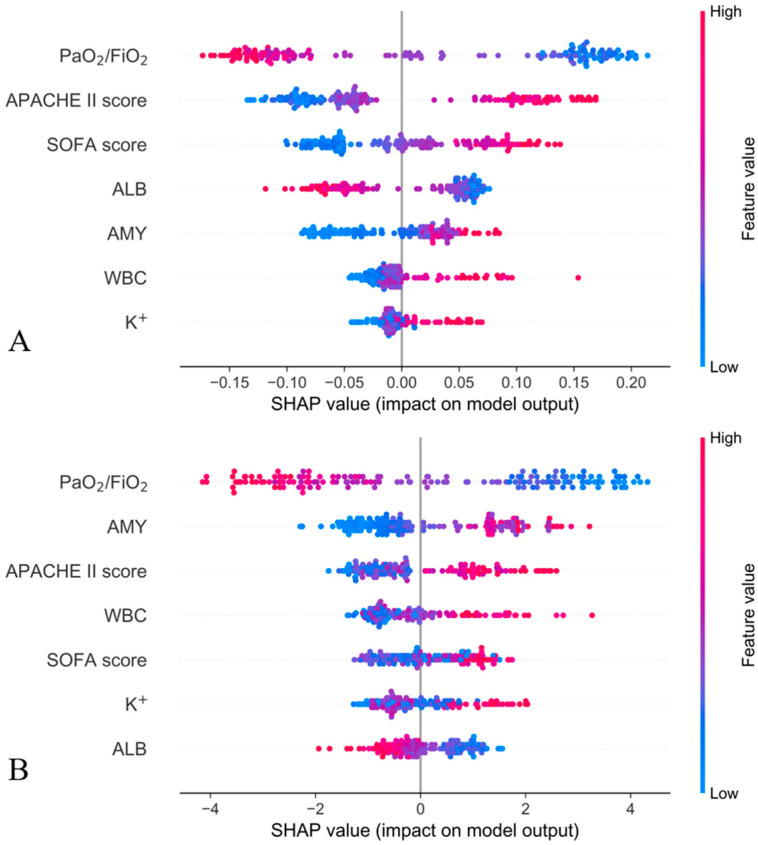
SHAP summary plot to determine the importance ranking of the seven features that have the greatest impact on the RF and XGB models. (**A**): SHAP summary plot of RF model; (**B**): SHAP summary plot of XGB model.

The SHAP summary plot combines the influence of feature importance and features to explain the relationship between features and models comprehensively. Each point in the plot represents a sample, with the position on the y-axis determined by the feature, and the position on the x-axis determined by the SHAP value of the feature. The colors represent the feature values from small to large, with redder colors indicating larger values and bluer colors indicating smaller values for the feature itself. The graph below (Figure 4A,B) shows that the PaO_2_/FiO_2_ is very important to the RF model and that a lower PaO_2_/FiO_2_ indicates a greater likelihood of ARDS.

After excluding the interference of other irrelevant features, and again using the SHAP summary plot, this work determined the order of importance of the seven features with the most significant impact on the RF and XGB models, respectively. The order of importance of the features in the RF model was: PaO_2_/FiO_2_, APACHE II, SOFA, ALB, AMY, WBC, K^+^; in the XGB model, the order of importance of the features was: PaO_2_/FiO_2_, AMY, APACHE II, WBC, SOFA, K^+^, ALB. In order to further reduce noise and optimize the model effect, the four most important features for predicting the risk of ARDS in SAP patients were selected by combining the ranking of feature importance and expert consensus: PaO_2_/FiO_2_, AMY, APACHE II, and SOFA. 

In addition, to facilitate the early assessment of the severity of ARDS in patients by clinicians, this work presents a quaternary classification study for the first time. In this modeling, the four characteristic variables obtained by screening were used to construct the model of this work. The models used in the quaternary classification study include RF, SVM, DT, XGB, and ANN. The newly added ANN model is a complex network structure formed by interconnecting a large number of processing units, which has been shown to provide a more accurate response to the probability of risk occurrence than general statistical methods. The predictive performance metrics of the five models in the test set are shown in Table 4. The comparison shows that the ANN model can predict ARDS severity with 86% accuracy, with 82%, 78%, and 86% precision predicting mild, moderate, and severe ARDS, 93%, 72%, and 95% recall, and 87%, 75%, and 90% F1 values, the best performance among the five models. XGB and RF are the following best models, with 81% and 77% prediction accuracy being the next best.

## 4. Discussion

ARDS is one of the most common complications in patients with SAP, with an average of one in every three patients complicated with ARDS [25,26]. In patients with acute onset of bilateral pulmonary edema accompanied by refractory hypoxemia, in-hospital mortality can reach more than 30% [27]. Therefore, early identification of patients at high risk of ARDS and prediction of the severity of the disease is essential. More than 20 scoring systems have been clinically developed to predict the deterioration of SAP patients, such as the RANSON and BISAP scores. However, the clinical utility of these scores still needs to be determined [28]. However, these prediction systems based on rule learning methods have limitations when dealing with complex data and complex data relationships, so clinical utility still needs to be determined [29]. Lung Injury Prediction Score (LIPS) is one of the most commonly used scores to predict ARDS in high-risk patients [30], but prospective follow-up shows that its positive predictive value is limited [31].

In recent years, machine learning has been increasingly applied to the medical field because of its accuracy, objectivity, and rapidity in improving the reliability and accuracy of diagnostic systems for specific diseases [32]. It is crucial in the prediction of disease. Therefore, this work attempts to establish an early prediction model for the occurrence and severity of ARDS in SAP patients by using available clinical objective data in combination with machine learning methods to help clinicians make rapid and accurate judgments on high-risk patients.

In this work, five machine learning algorithms, including LR, RF, XGB, SVM, and DT, are used to predict the occurrence of ARDS in SAP. The prediction effects of the models are excellent through the evaluation of some indicators. Among them, the XGB model has been proven to have the best all-around prediction performance because its AUC value reaches 0.93. Other indicators, such as accuracy and precision, are significantly better than the other four models. The XGB model is an optimized distributed gradient lifting library with high efficiency, flexibility, and portability advantages. Based on this, Le et al. [10] independently verified the value of the XGB model in the early prediction of ARDS. They found that the AUC value of the XGB model reached 0.827, 0.810, and 0.790, respectively, when tested for the detection of ARDS at 12 h, 24 h, and 48 h prior to onset. The AUC values obtained in the above studies were slightly lower than ours, which may be because the investigators did not optimize the characteristic variables. Noise in the data may lead to instability between the model and its predicted results, thereby reducing the generalization of its effects [24]. Another study that predicted the duration of mechanical ventilation in patients with ARDS also found that the XGB model stood out among many models and exhibited the most stable and accurate performance, called the “optimal model” by the authors [33].

The ANN algorithm is famous for its powerful object classification ability [22]. A retrospective work conducted by Yang et al. [34] also confirmed that the ANN model is a powerful tool for predicting ARDS after SAP. However, this work only included a small number of characteristic variables that the authors subjectively considered relevant, which may have overlooked some critical potential indicators and thus reduced the accuracy of the model. Therefore, in this work, five models, RF, XGB, SVM, DT, and ANN, were trained based on the critical features obtained by feature optimization in predicting ARDS severity. The systematic comparison shows that the ANN model can most accurately reflect the severity of ARDS compared with RF, XGB, SVM, and DT models, and its accuracy rate value is 86%. The XGB model, which has previously been excellent in predicting the occurrence of ARDS, occupies the second place with an accuracy rate of 81%.

In addition, this work explains the ranking of essential characteristic variables in the SHAP summary plot in combination with expert opinions. PaO_2_/FiO_2_ has the highest contribution value in the model. As one of the commonly used indicators for the diagnosis and severity grading of ARDS in clinical practice, studies have shown that dynamic monitoring of its changes can detect mild forms of ARDS in a timely manner, which helps to immediately start corresponding lung protective ventilation strategies and avoid treatment delay [27]. A study on high-risk patients with ARDS in the intensive care unit showed that plasma amylase was markedly increased, although in the absence of pancreatitis [35]. The relationship between plasma amylase and other lung diseases has also been confirmed. Its elevation was even related to the adverse clinical outcome of patients with coronavirus disease in 2019 [36]. This may be because a large amount of pancreatic amylase can cause pulmonary vasoconstriction by activating the relevant complement and promoting the release of bioactive substances such as histamine, which leads to pulmonary dysfunction [37]. APACHE II and SOFA scores have been proven to be effective in reflecting the dynamic changes in the clinical condition of critical patients and have high predictive value [38]. They are widely used in predicting the secondary multiple organ failure of SAP patients, which has received high evaluation [39]. In addition, ALB and WBC levels also play a role in the accurate prediction model. Ocskay. K. et al. [40] found that about 1/5 AP patients were accompanied at admission, and severe hypoproteinemia was independently related to subsequent organ failure. In systemic inflammation, the production of albumin may be reduced to make room for the production of proinflammatory cytokines [41]. Elevated proinflammatory cytokines such as IL-6 and IL-8 are associated with lung injury [27]. The elevation of leukocytes due to severe inflammation during SAP can cause an increase in inflammatory mediators through a variety of signal transduction mechanisms and induce the occurrence and development of ARDS [42].

The strength of this work is that the characteristic variables are all taken from routine clinical tests, so there is no need to waste costs collecting additional data. Secondly, the noise reduction is repeated in the optimization process of the characteristic variables of the dataset, which makes the sample characteristics clearer and improves the model prediction effect. As confirmed by the AUC value and other evaluation indicators, the effects of the five models after optimization are significantly improved. More importantly, our study takes the lead in predicting the severity of ARDS in SAP patients. The prediction accuracy of the trained ANN model can reach 86%, which facilitates doctors to implement different degrees of respiratory support strategies for high-risk patients with ARDS as early as possible. It can prevent further deterioration of the patient’s condition, which has significant clinical application value.

It must be acknowledged that this research has certain limitations. First, this is a single-center retrospective study, which may have some selection bias. A multicenter prospective study must further verify the prediction effect of subsequent related models. Secondly, some new biomarkers (such as Ang-2, sRAGE, and cytokines) [43] are not included in our prediction indicators because of the high cost and failure to be widely detected clinically, which may limit the prediction effect of the model. Even so, the prediction models proposed in this study can help clinicians to predict early and judge the severity of ARDS in SAP.

## 5. Conclusions

In summary, this work screened four important indicators by machine learning methods. It developed relevant predictive models for predicting the risk of developing ARDS in SAP patients, and the models were shown to be excellent. Meanwhile, this work is the first quaternary classification work to establish five models for early assessment of ARDS severity, and the accuracy of the ANN model reached 86%, which suggests the potential value of machine learning models in predicting complications in SAP patients. This work combines machine learning with SHAP, which can also promote the optimization of the model while explaining the model in depth. This model can also be applied in the risk prediction of other diseases and provide better explanations. This work will consider expanding the amount of data through multi-center research in the future to adjust the optimization model further. 

## Figures and Tables

**Table 1:** The baseline characteristics of ARDS and non-ARDS groups.

Variables	Patients (*N* = 440)	Non–ARDS (*N* = 230)	ARDS (*N* = 210)	*p* Value
Demographic data				
Age (year)	52.00 (41.00–71.00)	54.00 (40.75–73.00)	52.00 (41.00–67.00)	0.550
Sex (male)	284/440 (64.5%)	155/230 (67.4%)	129/210 (61.7%)	0.566
smoking	141/440 (32.0%)	70/230 (30.4%)	71/210 (34.0%)	0.710
Alcohol intake	131/440 (29.8%)	60/230 (26.1%)	71/210 (34.0%)	0.403
Hypertension	189/440 (43.0%)	100/230 (43.4%)	89/210 (42.6%)	0.928
Diabetes	110/440 (25.0%)	70/230 (30.4%)	40/210 (19.1%)	0.207
Coronary heart disease	28/440 (6.4%)	15/230 (6.5%)	13/210 (6.4%)	0.978
Etiology				0.912
Biliary	241/440 (54.8%)	120/230 (52.2%)	121/210 (57.4%)	
Hypertrigly-ceridemia	156/440 (35.5%)	85/230 (40.0%)	71/210 (34.0%)	
Alcoholic	15/440 (3.4%)	15/230 (6.5%)	0/210 (0%)	
Others	14/440 (3.2%)	10/230 (4.3%)	4/210 (2.1%)	
scoring system for disease severity				
APACHE II	9.00 (6.50–15.00)	7.50 (5.00–10.00)	13.00 (9.00–16.00)	<0.05
SOFA	3.00 (1.50–5.00)	2.00 (1.00–3.00)	4.00 (2.00–7.00)	<0.05
Laboratory index				
SBP	129.00 (116.00–142.00)	129.00 (115.50–139.25)	128.00 (116.00–147.00)	0.423
DBP	78.00 (70.00–86.00)	78.00 (70.00–85.30)	78.00 (70.00–87.00)	0.234
MAP	95.00 (87.30–105.50)	94.80 (87.30–105.30)	96.00 (86.70–106.70)	0.259
WBC	15.00 (11.30–16.80)	15.10 (9.70–16.60)	14.70 (12.70–19.10)	<0.05
PLT	177.00 (143.00–227.00)	188.5 (145.80–216.80)	171.0 (138.00–259.00)	0.243
HGB	140.00 (127.50–165.50)	140.00 (127.80–165.30)	137.00 (127.00–167.00)	0.888
NEUT%	88.40 (84.70–91.50)	88.15 (84.18–91.33)	88.90 (84.81–91.64)	0.889
HCT	40.10 (34.40–44.20)	40.50 (37.50–44.00)	39.70 (33.90–44.60)	0.161
PCT	1.16 (0.33–4.96)	0.75 (0.26–2.55)	1.39 (0.35–6.49)	<0.05
CRP	167.00 (85.30–252.00)	126.00 (48.40–228.00)	179.90 (132.50–271.80)	<0.05
BUN	7.00 (4.70–9.40)	6.00 (4.60–8.70)	7.30 (5.30–10.00)	<0.05
UA	328.00 (207.00–416.75)	329.00 (204.00–390.00)	327.00 (216.00–448.00)	<0.05
TG	2.82 (1.16–7.94)	2.22 (1.05–6.20)	3.16 (1.38–18.87)	0.090
CHOL	4.17 (3.15–6.46)	4.17 (3.66–6.41)	4.14 (3.02–6.58)	0.845
ALB	34.00 (31.15–37.80)	35.55 (31.53–39.35)	33.00 (30.60–35.00)	0.366
TBIL	21.25 (15.26–29.68)	20.75 (14.35–29.93)	25.15 (16.70–30.23)	0.382
DBIL	9.10 (4.70–19.95)	8.20 (4.50–16.40)	10.40 (6.10–21.20)	0.295
UBIL	11.30 (8.70–16.35)	11.30 (8.60–14.90)	11.20 (8.70–17.60)	0.900
AST	36.00 (25.50–71.50)	33.00 (25.75–62.75)	42.00 (25.00–79.00)	0.091
ALT	29.00 (17.00–74.00)	37.00 (20.00–84.25)	26.00 (15.00–64.00)	0.153
γ-GTT	71.00 (36.50–198.50)	87.00 (49.00–223.75)	66.00 (32.00–158.00)	0.274
ALP	79.00 (63.00–117.00)	79.00 (62.00–114.00)	80.00 (63.00–119.00)	0.234
PaO_2_/FiO_2_	271.10 (213.15–373.35)	331.00 (270.90–445.80)	227.00 (173.10–289.90)	<0.05
Lac	1.80 (1.00–2.55)	1.80 (1.10–2.40)	1.80 (0.90–2.70)	0.104
K^+^	3.92 (3.58–4.35)	3.91 (3.58–4.26)	4.03 (3.56–4.71)	<0.05
Ca^2+^	1.12 (1.02–1.17)	1.14 (1.06–1.18)	1.08 (0.94–1.15)	0.522
Na^+^	138.40 (134.80–140.70)	138.50 (135.90–141.00)	137.50 (134.60–140.50)	0.479
Cl^−^	109.00 (106.50–111.60)	108.80 (106.70–111.20)	109.40 (105.40–112.30)	0.556
HCO_3_^−^	20.20 (17.30–23.20)	20.60 (18.40–23.10)	19.60 (16.30–23.30)	<0.05
BE	−3.65 (−6.65–−1.55)	−3.30 (−5.40–−1.30)	−3.70 (−7.00–−1.70)	<0.05
Gap	9.00 (6.80–11.33)	9.10 (7.40–11.45)	8.90 (5.50–11.10)	0.199
AMY	724.00 (261.50–1474.00)	412.00 (206.00–985.50)	954.00 (356.00–2000.00)	<0.05
PT	13.10 (12.05–14.25)	13.25 (12.15–14.58)	12.80 (12.00–14.10)	0.175
APTT	13.10 (12.05–14.25)	29.70 (27.58–32.32)	30.20 (27.10–35.20)	<0.05
DDHS	1480.50 (805.50–2805.75)	1301.00 (479.00–3040.00)	1877.00 (1065.00–2722.00)	0.101

Non–ARDS = patients without acute respiratory distress syndrome, ARDS = patients with acute respiratory distress syndrome, Acute Physiology and Chronic Health Evaluation (APACHE II), Sequential Organ Failure Assessment (SOFA), systolic blood pressure (SBP), diastolic blood pressure (DBP), mean arterial pressure (MAP), White blood cell count (WBC), neutrophil ratio (NEUT%), platelet count (PLT), hemoglobin count (HGB), hematocrit (HCT), procalcitonin (PCT), and high-sensitivity C-reactive protein (CRP), Blood urea nitrogen (BUN), blood uric acid (UA), triglyceride (TG), cholesterol (CHOL), albumin (ALB), potassium (K^+^), calcium (Ca^2+^), sodium (Na^+^), chloride (Cl^−^), bicarbonate ion (HCO_3_^−^), blood amylase (AMY), total bilirubin (TBIL), direct bilirubin (DBIL), indirect bilirubin (UBIL), glutamic-pyruvate transaminase (ALT), glutamic-oxaloacetate transaminase (AST), gamma-glutamyl transpeptidase (γ-GGT), alkaline phosphatase (ALP), prothrombin time (PT), activated partial thromboplastin time (APTT), D-dimer (DDHS), oxygenation index (PaO_2_/FiO_2_), lactate (Lac), base excess (BE), arterial-venous carbon dioxide partial pressure difference (Gap).

**Table 2:** The predictive value of five models for the occurrence of ARDS in SAP patients.

	Accuracy	Precision	Recall	F1 Score	AUC
**LR**	0.78	0.81	0.74	0.77	0.76
**RF**	0.81	0.87	0.73	0.79	0.81
**DT**	0.65	0.64	0.71	0.67	0.65
**SVM**	0.78	0.84	0.69	0.76	0.80
**XGB**	0.84	0.86	0.81	0.83	0.84

Logical Regression (LR), Random Forest (RF), Decision Tree (DT), Support Vector Machine (SVM), and eXtreme Gradient Boosting (XGB).

**Table 3:** Comparison of the prediction performance of the five models before and after optimization.

	AUC	Improve	Accuracy	Improve	Precision	Improve	Recall	Improve	F1 Score	Improve
**LR**	0.85	9%	0.85	7%	0.83	2%	0.89	15%	0.86	9%
**RF**	0.87	6%	0.87	6%	0.90	3%	0.85	12%	0.88	9%
**DT**	0.85	20%	0.85	20%	0.83	19%	0.89	18%	0.86	19%
**SVM**	0.86	6%	0.87	9%	0.86	2%	0.89	20%	0.87	11%
**XGB**	0.93	9%	0.93	9%	0.92	6%	0.95	14%	0.93	10%

**Table 4:** Predictive efficacy of the five models in the severity of ARDS.

	Accuracy				Precision				Recall				F1-Score			
	Non	Mild	Moderate	Severe	Non	Mild	Moderate	Severe	Non	Mild	Moderate	Severe	Non	Mild	Moderate	Severe
RF	0.77				0.81	0.90	0.70	0.71	0.85	0.60	0.65	0.85	0.83	0.72	0.68	0.77
XGB	0.81				0.82	0.92	0.77	0.71	0.88	0.80	0.68	0.75	0.85	0.86	0.72	0.73
SVM	0.70				0.71	0.75	0.77	0.50	0.77	0.63	0.57	0.30	0.81	0.69	0.66	0.37
DT	0.75				0.82	0.75	0.63	0.74	0.85	0.60	0.65	0.70	0.83	0.67	0.64	0.72
ANN	0.86				0.90	0.82	0.78	0.86	0.89	0.93	0.72	0.95	0.90	0.87	0.75	0.90

Random Forest (RF), Decision Tree (DT), Support Vector Machine (SVM), and eXtreme Gradient Boosting (XGB), Artificial Neural Network (ANN).

## Data Availability

All data generated or analysed during this study are included in this published article.
